# Culture Condition of Bone Marrow Stromal Cells Affects Quantity and Quality of the Extracellular Vesicles

**DOI:** 10.3390/ijms23031017

**Published:** 2022-01-18

**Authors:** Amanda L. Scheiber, Cierra A. Clark, Takashi Kaito, Masahiro Iwamoto, Edwin M. Horwitz, Yuka Imamura Kawasawa, Satoru Otsuru

**Affiliations:** 1 Department of Orthopaedics, University of Maryland School of Medicine, Baltimore, MD 21201, USA; ascheiber@som.umaryland.edu (A.L.S.); caclark806@gmail.com (C.A.C.); masahiro.iwamoto@som.umaryland.edu (M.I.); 2 Department of Orthopaedic Surgery, Graduate School of Medicine, Osaka University, Osaka 565-0871, Japan; takashikaito@ort.med.osaka-u.ac.jp; 3 Aflac Cancer and Blood Disorders Center, Children’s Healthcare of Atlanta and Emory University Department of Pediatrics, Atlanta, GA 30322, USA; edwin.horwitz@emory.edu; 4 Departments of Pharmacology and Biochemistry and Molecular Biology, Institute of Personalized Medicine, Penn State University, College of Medicine, Hershey, PA 17033, USA; yimamura@pennstatehealth.psu.edu

**Keywords:** bone marrow stromal cells (BMSCs), extracellular vesicles (EVs), miRNAs, culture condition

## Abstract

Extracellular vesicles (EVs) released by bone marrow stromal cells (BMSCs) have been shown to act as a transporter of bioactive molecules such as RNAs and proteins in the therapeutic actions of BMSCs in various diseases. Although EV therapy holds great promise to be a safer cell-free therapy overcoming issues related to cell therapy, manufacturing processes that offer scalable and reproducible EV production have not been established. Robust and scalable BMSC manufacturing methods have been shown to enhance EV production; however, the effects on EV quality remain less studied. Here, using human BMSCs isolated from nine healthy donors, we examined the effects of high-performance culture media that can rapidly expand BMSCs on EV production and quality in comparison with the conventional culture medium. We found significantly increased EV production from BMSCs cultured in the high-performance media without altering their multipotency and immunophenotypes. RNA sequencing revealed that RNA contents in EVs from high-performance media were significantly reduced with altered profiles of microRNA enriched in those related to cellular growth and proliferation in the pathway analysis. Given that pre-clinical studies at the laboratory scale often use the conventional medium, these findings could account for the discrepancy in outcomes between pre-clinical and clinical studies. Therefore, this study highlights the importance of selecting proper culture conditions for scalable and reproducible EV manufacturing.

## 1. Introduction

Bone marrow stromal cells (BMSCs) including so-called mesenchymal stem/stromal cells have been utilized in cell therapy for various diseases and have demonstrated the potency to provide beneficial effects both clinically and pre-clinically [1]. To this day, there are over 1500 clinical trials using mesenchymal stem/stromal cells underway in the world according to ClinicalTrials.gov (accessed on 3 January 2022). Since BMSCs contain multipotent stem cells, they have been expected to replace tissues by differentiating into the tissue cells. However, accumulating data suggest that BMSCs exert their therapeutic effects in a paracrine manner by secreting growth factors and cytokines as well as releasing extracellular vesicles (EVs) [2,3,4,5].

EVs are membrane-enclosed small vesicles ranging from 30 nm to 1000 nm in diameter that contain bioactive molecules such as growth factors, cytokines, mRNA, and micro RNA (miRNA) [6]. EVs play an important role in intercellular communication by transferring the bioactive contents between cells [7,8,9]. Cargos in EVs delivered from one cell to another become active and functionally alter the recipient [10,11].

Indeed, EVs released from BMSCs also contain biological molecules and mimic the beneficial effects of BMSCs in various disease conditions including myocardial infarction [12], pulmonary hypertension [13,14], kidney injury [15], brain injury [16], muscle injury [17], skin wound [18], and fractures [19]. This suggests that BMSC-derived EVs hold great promise to be a critical mediator of the therapeutic activity of BMSCs and may substitute for BMSCs as cell-free therapy. Although no significant adverse events including malignant transformation have been reported from clinical trials of BMSC [20,21,22,23], cell-free therapies using EVs could possess fewer safety concerns as they lack the ability to transform to malignant cells. Moreover, BMSCs freshly thawed from cryopreservation fail to exert therapeutic effects and require culture incubation to recover the effects, which restricts their application and accessibility [24,25,26,27]. Since EVs are relatively stable and can be cryopreserved without losing their activity [28,29], cell-free therapy with EVs offers potential advantages over cell therapy with BMSCs.

Despite the promising potential of EVs for therapeutic applications, one of the current challenges toward clinical translation is to establish scalable EV manufacturing processes with consistently reproducible quality [5,30]. Larger-scale EV production can be accomplished by simply employing higher quantities of the parental cells. For the clinical application of cell therapy, various culture systems to manufacture scalable BMSCs have been developed. These include static conventional 2D culture using multilayer culture flasks and dynamic 3D culture with various types of bioreactors in combination with culture medium optimized for cell expansion (high-performance medium) [31,32,33,34]. BMSCs cultured in these scalable platforms have been demonstrated to be rapidly expanded without altering their immunophenotype and multipotency [33], suggesting the promising potential for scalable EV production. Indeed, BMSCs expanded in a bioreactor with a high-performance medium have been reported to produce significantly more EVs compared to conventional 2D culture [35,36]. However, despite the increased EV production, the influence of the scalable expansion of BMSCs to EV quality has not been fully elucidated. Given that culture condition is thought to affect EV quality [37,38,39], EVs derived from rapidly expanded BMSCs could be altered both in quantity and quality.

Therefore, in this study, using a conventional 2D culture system excluding the effects of dynamic shear forces from a bioreactor system, we sought to examine the effects of high-performance media on EV production from BMSCs isolated from nine healthy donors. We characterized EVs by nanoparticle tracking analysis and compared the EV production rate of BMSCs cultured in different culture media. We further investigated the impact of culture media on cargos packaged in EVs, specifically focusing on microRNAs contained in EVs. Overall, culturing the same BMSCs in different culture media excludes the variances from donors and allows us to determine the effects of culture media on the quantity and quality of EVs released from BMSCs, highlighting the importance of selecting the culture medium for optimal EV production for the target diseases.

## 2. Results

### 2.1. Accelerated Proliferation of BMSCs Cultured in High-Performance Media

**Figure 1:**
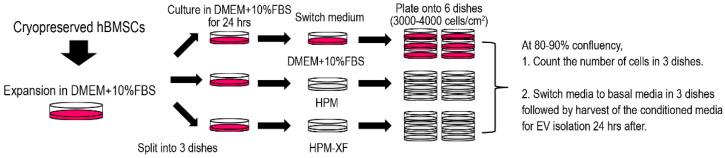
Schematic workflow of human BMSC culture for EV isolation and analyses. Cryopreserved human BMSCs were expanded in DMEM supplemented with 10%FBS (DMEM + 10%FBS) and evenly split into 3 dishes. After 24 h incubation in DMEM + 10%FBS, the medium was switched to either fresh DMEM + 10%FBS, HPM, or HPM-XF. At 80–90% confluency, cells were re-plated onto 6 dishes at the density of 3000–4000 cells/cm^2^. At 80–90% confluency, the number of cells was counted in 3 dishes out of 6. In the rest dishes, the media were aspirated and replaced with basal media after a thorough wash with PBS (3 times). The conditioned media were harvested after 24 h of incubation for EV analysis.

**Figure 2:**
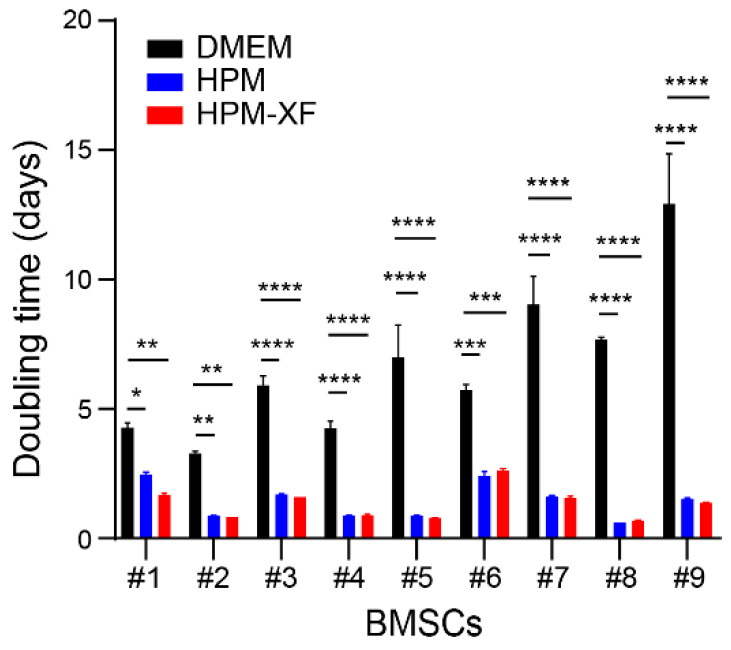
Doubling time. Doubling times were calculated from the number of cells at the beginning of culture and the harvest as well as the culture duration (days). Doubling times of each BMSC in three different media (DMEM, HPM, and HPM-XF) were presented as mean ± SEM. * *p* < 0.05, ** *p* < 0.01, *** *p* < 0.001, and **** *p* < 0.0001. Black bar: BMSCs cultured in DMEM + 10%FBS. Blue bar: BMSCs cultured in HPM. Red bar: BMSCs cultured in HPM-XF.

Cryopreserved BMSCs, that were previously established from the bone marrow of nine healthy donors and expanded in DMEM culture medium, were thawed and recovered in DMEM culture medium as shown in Figure 1 [40]. To determine if the proliferation of these BMSCs expanded in DMEM culture medium can be accelerated by commercially available high-performance culture media, each BMSC line was split into three dishes and cultured in either DMEM culture medium, high-performance medium (HPM), or xeno-free high-performance medium (HPM-XF). Once expanded, BMSCs were plated at the density of 3000–4000 cells /cm^2^ and cultured in each medium. At 80–90% confluency, the number of cells was counted, and the doubling time was calculated based on the cell number at the beginning and the end as well as the days in culture. The doubling time of BMSCs widely varied between donors. However, it was significantly reduced when BMSCs from each donor were cultured in HPM and HPM-XF compared to when they were in DMEM (Figure 2). These results indicate that HPM and HPM-XF are able to accelerate the proliferation of BMSCs even though these BMSCs were originally established in DMEM.

### 2.2. Characterization of BMSCs Expanded in DMEM and High-Performance Media

**Figure 3:**
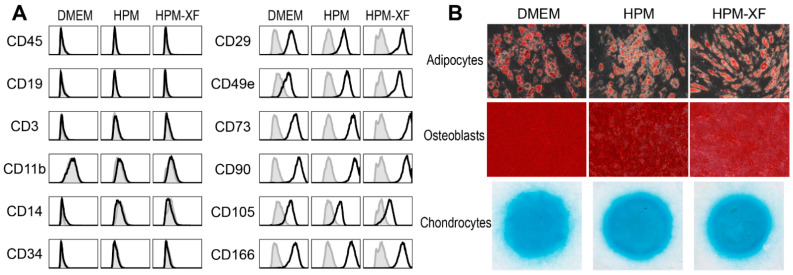
Characterization of BMSCs cultured in three different media (DMEM, HPM, and HPM-XF). (**A**) Flow cytometric analysis for hematopoietic markers and mesenchymal markers. Histograms of experimental samples (black solid line) were overlaid with the isotype control (filled with gray). (**B**) BMSCs were differentiated into adipocytes, osteoblasts, and chondrocytes and stained with Oil Red O, Alizarin Red, and Alcian Blue, respectively. Data from one representative sample are presented.

To determine whether HPM and HPM-XF alter characteristics of BMSCs, BMSCs expanded in each medium were harvested and characterized their phenotypes by flow cytometry. The expanded BMSCs in all three media expressed mesenchymal surface markers such as CD29, CD49e, CD73, CD90, CD105, and CD166, while lacking the expression of hematopoietic markers such as CD3, CD11b, CD14, CD19, CD34, and CD45. The expression patterns of surface markers were phenotypically indistinguishable between BMSCs expanded in these three culture media (Figure 3A).

To examine the in vitro multipotency, these BMSCs were cultured in adipogenic, osteogenic, and chondrogenic differentiation media. The successful differentiation into adipocytes, osteoblasts, and chondrocytes were demonstrated by oil red O staining, Alizarin red staining, and Alcian blue staining, respectively (Figure 3B).

These characteristics of BMSCs met the minimal criteria of mesenchymal stromal cells proposed by the International Society for Cell & Gene Therapy (ISCT) [41,42]. Regardless of culture media, all BMSCs were comparable to each other and preserved the characteristics of mesenchymal stromal cells, suggesting that, despite the acceleration in proliferative activity, neither HPM nor HPM-XF transformed BMSCs.

### 2.3. Enhanced EV Production from BMSCs in High-Performance Media

**Figure 4:**
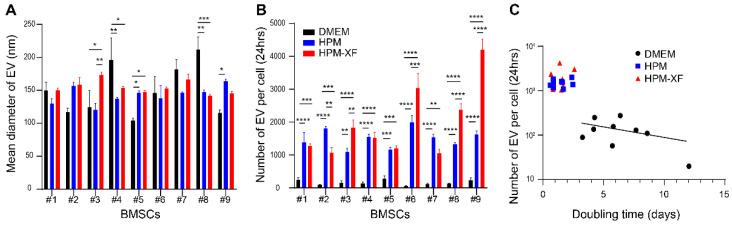
Characterization of EVs harvested from BMSC cultures. (**A**) The average size of EVs was evaluated by nanoparticle tracking analysis (NanoSight). (**B**) The number of EVs released from a single BMSC was calculated from the total number of EVs in the conditioned medium and the number of BMSCs in the culture. (**C**) The number of EVs released from a single BMSC over the doubling time of the BMSC was plotted. The trend line for EVs from DMEM was shown. Results are shown as mean ± SEM. * *p* < 0.05, ** *p* < 0.01, *** *p* < 0.001, and **** *p* < 0.0001. Black: EVs from BMSCs cultured in DMEM + 10%FBS. Blue: EVs from BMSCs cultured in HPM. Red: EVs from BMSCs cultured in HPM-XF.

We then examined whether EV production from BMSCs is affected by culture media. At 80–90% confluency, BMSCs were washed with PBS three times and the culture media were replaced with basal media which do not contain FBS, growth factors, or cytokines. After 24 h of incubation at 37 °C, the conditioned media were harvested and proceeded with centrifugation and filtration followed by NanoSight analysis. The average EV size produced by BMSCs was mostly in the range of 100 to 200 nm. Although statistical significance was detected in EV size between media in some of BMSCs, most of them remained within the range (Figure 4A). The number of EVs produced per BMSC during the 24 h was calculated from the total number of EVs in the conditioned media and the total number of cells per dish. In all BMSCs, the EV production was significantly enhanced when they were cultured in HPM or HPM-XF compared to in DMEM (Figure 4B). BMSCs produced averaged 13- and 16-fold EVs in HPM and HPM-XF over DMEM, respectively. When the number of EVs produced per BMSC during the 24 h was plotted with the BMSC doubling time, there was a general trend that BMSCs with shorter doubling time produced more EVs (Figure 4C). Of note, this trend was observed even in the BMSCs cultured in DMEM, suggesting that EV production is somehow correlated to proliferation rate shown as the trend line in Figure 4C. These findings demonstrated that EV production from BMSCs can be enhanced by using HPM or HPM-XF.

### 2.4. RNA Contents in EVs from BMSCs

The contents of EVs such as RNAs and proteins are thought to be critical for their therapeutic actions [43,44]. Thus, using RNA as an example, we evaluated the contents of EVs from BMSCs cultured in different media to determine if culture media affect EV quality in addition to quantity. In the following experiments, we focused on six lines of BMSCs (#3–#8) cultured in DMEM and HPM.

**Figure 5:**
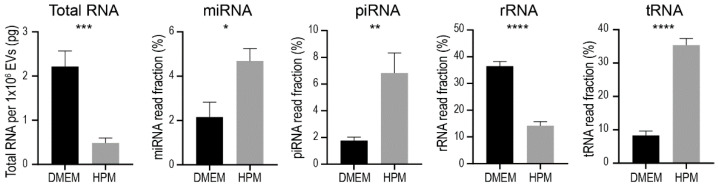
Fraction of RNA species contained in EVs from BMSCs cultured in either DMEM + 10%FBS (black bar) or HPM (gray bar). Data are shown as mean ± SEM. * *p* < 0.05, ** *p* < 0.01, *** *p* < 0.001, and **** *p* < 0.0001.

After the NanoSight analysis for EV concentration, EVs were isolated from the BMSC conditioned media by ultracentrifugation followed by RNA isolation. RNA quantity and quality were examined with an Agilent 2100 Bioanalyzer followed by small RNA sequencing. Interestingly, the total amount of RNA contained in 1 × 10^6^ EVs was significantly less in EVs from BMSCs cultured in HPM compared to those from BMSCs cultured in DMEM, although BMSCs cultured in HPM produced significantly more EVs (Figure 5). RNA sequencing revealed that fractions of RNA species were significantly different between EVs from BMSCs cultured in DMEM and HPM (Figure 5). For instance, fractions of miRNA, piRNA, tRNA were significantly higher in EVs from BMSCs cultured in HPM, while rRNA was opposite (Figure 5). These results suggest that the contents packaged in EVs are affected by BMSC culture media even though the phenotypes and differentiation ability of BMSCs are not substantially altered.

### 2.5. Profiling miRNA in EVs from BMSCs

**Figure 6:**
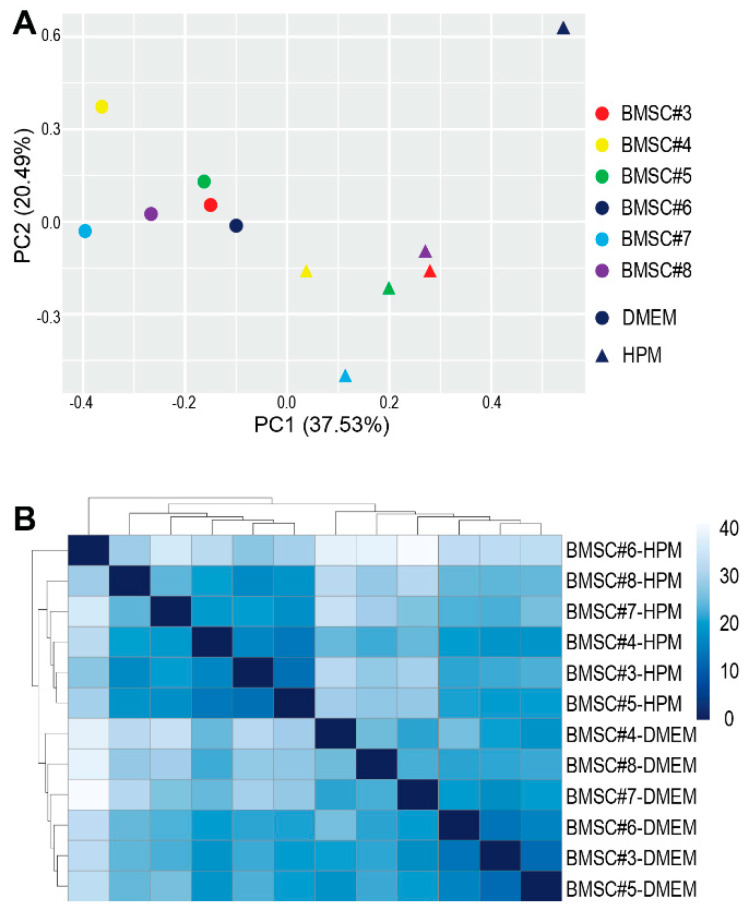
Analysis of miRNA profiles. (**A**) Principal component analysis of miRNA profiles of each EV sample isolated from BMSCs (#3–#8) cultured in either DMEM + 10%FBS (●) or HPM (▲). (**B**) Heat map and hierarchical clustering analysis of each EV sample isolated from BMSCs (#3-#8) cultured in either DMEM + 10% or HPM. The sequencing data are deposited in the GEO (the accession number: GSE185942).

To qualitatively assess the contents in EVs, miRNA transcriptome analysis was performed by small RNA sequencing. Principal component analysis of miRNA transcriptome in EVs from BMSCs #3 to #8 revealed that EVs from BMSCs cultured in DMEM were clustered separately from those from BMSCs in HPM although miRNA profiles were different from donor to donor (Figure 6A). A heat map and hierarchical clustering analysis further confirmed that different culture media provided a vaster diversity of miRNAs in EVs than the donor difference (Figure 6B). These results demonstrate that culture media have greater impacts not only on EV quantity but also on EV quality.

### 2.6. Upregulation of miRNAs Related to Cellular Growth and Proliferation

**Figure 7:**
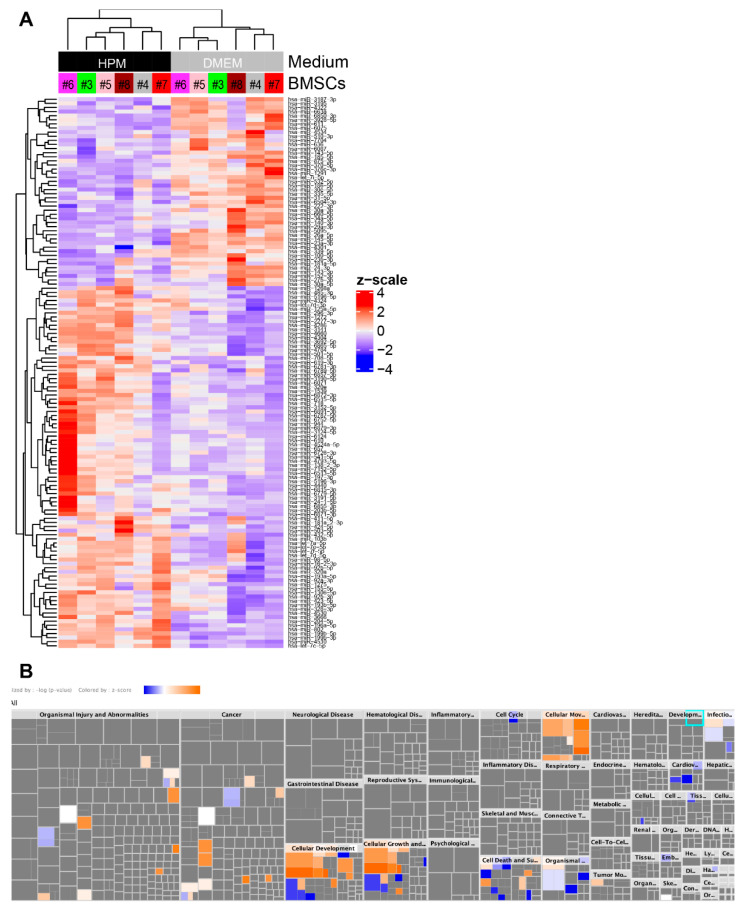
Differential gene expression analysis and ingenuity pathway analysis. (**A**) Heat map and hierarchical clustering analysis shows miRNAs upregulated (88 miRNAs) and downregulated (46 miRNAs) in EVs from BMSCs cultured in HPM compared to those from BMSCs cultured in DMEM + 10%FBS. (**B**) Ingenuity pathway analysis for the diseases & functions of 88 upregulated and 46 downregulated miRNAs identified in (**A**).

To characterize alterations in miRNA profiles, differential gene expression analysis was performed between EVs from BMSCs cultured in DMEM and HPM. We identified 88 miRNAs upregulated and 46 miRNAs downregulated in EVs from BMSCs cultured in HPM compared to those from BMSCs cultured in DMEM (Figure 7A). Ingenuity pathway analysis (IPA) revealed that these differentially expressed miRNAs were mainly enriched in “Cellular Development”, “Cellular Growth and Proliferation”, “Cellular Movement”, and “Cell Death and Survival” (Figure 7B). Given that HPM accelerates BMSC proliferation, these results suggest that the changes in miRNA transcriptomes may reflect the cellular status in culture affected by culture media.

## 3. Discussion

EVs released from BMSCs have been demonstrated to play an important role in their therapeutic actions for various diseases. It is expected that EVs can be a substitution for BMSCs and lead to developing cell-free therapy that can bypass potential issues related to cell therapy such as malignant transformation [45]. However, the reliability of EV therapy depends on the quality and reproducibility of EV manufacturing, as the same challenges exist in cell therapy using BMSCs [5,46]. Additionally, similar to cell therapy, a larger number of EVs are required for clinical use compared to animal studies. Therefore, scalable and reproducible EV manufacturing processes need to be developed for the successful implementation of EV therapy for patients.

Recent advance in cell culture technologies has made great progress in achieving scalable production of BMSCs. Various types of bioreactors that culture cells three-dimensionally have been shown to achieve rapid expansion of BMSCs without altering their multipotency and immunophenotypes [33]. Of interest, it has been demonstrated that BMSCs expanded in these 3D culture systems produce a significantly larger amount of EVs [35,36,47], although the effects of bioprocess forces such as shear stress generated during 3D culture on the EV quality have not been fully elucidated [30,37]. In addition to culture systems, evolution has been made to culture media to allow rapid expansion of BMSCs without xenogeneic supplements optimized for clinical use [48]. These HPMs support the rapid expansion of BMSCs both in 2D and 3D culture systems. However, their effects on EV production and EV quality have not been fully explicated. Given that alterations in culture condition such as serum depletion, cell stress, and cell density affect the quantity and quality of EVs [37,38,39,49,50,51], it is critical to determine if the rapid expansion of BMSCs induced by HPM results in alteration of EV quantity and quality to develop scalable and reproducible EV manufacturing processes. Therefore, in this study, we examined the EV quantity and quality released from BMSCs cultured in HPM in comparison with those in the conventional DMEM culture medium. We found that all nine BMSCs tested in this study exhibited significant acceleration of proliferation by culturing in HPM or HPM-XF without losing their multipotency and immunophenotypes. EV analysis showed that both HPM and HPM-XF remarkably enhanced EV production. Interestingly, HPM significantly reduced RNA contents in EVs and altered the proportion of RNA species in EVs. Moreover, HPM-enriched miRNAs related to “Cellular Growth and Proliferation” in EVs, likely reflecting the proliferating condition of the parental BMSCs. Our results indicate that scalable EV manufacture can be achieved with HPM although it is not reproducing EVs from the conventional DMEM culture medium. Indeed, small RNA sequencing identified 88 upregulated miRNAs and 46 downregulated miRNAs in EVs from HPM culture compared to the DMEM culture. These 88 upregulated miRNAs included miRNAs that have been reported to suppress/inhibit tumor growth and metastasis such as miR-16-2-3p, miR-92b-3p, miR-197-3p, miR-204-5p, miR-320a, miR-411-5p, miR-424-5p, miR-708-5p, miR-718, miR-944, let-7a-5p, and let-7e-5p [52,53,54,55,56,57,58,59,60,61,62,63,64], suggesting that EVs from HPM culture may have superior anti-tumor effects. On the other hand, miRNAs known to protect articular cartilage and enhance cartilage regeneration such as miR-23a-3p, miR-26a-5p, miR-31-5p, miR-100-5p, and miR-140-3p are included in the 46 downregulated miRNAs [44,65,66,67,68], suggesting that EVs from HPM culture may have less therapeutic effects in the treatment for osteoarthritis. These findings indicate that different culture conditions likely produce EVs with different therapeutic effects. Given that pre-clinical studies performed at laboratory scale often use 2D culture with the conventional medium while scalable manufacturing processes are preferred for clinical usage, the difference in EV therapeutic potential could account for the discrepancy in outcomes between pre-clinical and clinical studies. Therefore, careful consideration of culture conditions needs to be taken especially when translating outcomes from pre-clinical studies to patients.

In this study, we focused on the miRNA profiles as one of the therapeutic components within EVs. Based on previous studies showing that culture condition alters protein contents in EVs [37,39,50,51], it reasonably follows that rapid expansion condition with HPM could affect other bioactive molecules. The detailed mechanisms underlying the alternation of contents by culture conditions remain unknown and need to be identified in the future. If identified, we might be able to modify the culture conditions to maintain high EV production without substantially changing the contents. Given that our results show that culture condition overcomes donor variance, optimizing culture condition may lead to reproducible EV production from BMSCs regardless of donors.

The comprehensive mechanisms of EV therapy have not been fully understood. Once bioactive molecules responsible for therapeutic action in each disease are identified, it may enable us to engineer EVs containing necessary molecules specific to each disease. This will be the next-generation cell-free therapy that can bypass the current issues with EVs such as reproducibility and consistency.

## 4. Materials and Methods

### 4.1. Cell Culture

A schematic workflow of bone marrow stromal cell (BMSC) culture for EV isolation and analyses is shown in Figure 1. Specifically, cryopreserved BMSCs at less than passage 3 which were previously isolated from healthy donors using density centrifugation and expanded in Dulbecco’s modified Eagle’s medium (DMEM; Corning, Glendale, AZ, USA) culture medium supplemented with 10% fetal bovine serum (FBS; Gemini Bio Products) were thawed and expanded again on a CellBIND culture dish (Corning) in the DMEM culture medium [26,40]. These BMSCs have been de-identified and the IRB at the University of Maryland School of Medicine exempted this study from human subject projects. When the cultures attained approximately 80–90% confluency, the cells were harvested by treating with 0.25% trypsin solution for 5 min at 37 °C and split into three dishes. After 24 h incubation in the DMEM culture medium, the medium in these 3 dishes was replaced with either the DMEM culture medium, the high-performance culture medium (HPM; RoosterNourish-MSC; KT-001, RoosterBio, Frederick, MD, USA), or the high-performance xeno-free culture medium (HPM-XF; RoosterNourish-MSC-XF; KT-016, RoosterBio). Once reached 80–90% confluency, the cells were harvested and plated onto 6 dishes at a density of 3000–4000 cells/cm^2^. At 80–90% confluency, 3 dishes were used to count the number of cells. The other 3 dishes were washed with phosphate-buffered saline (PBS) three times and replaced the media with basal media. After 24 h incubation, the conditioned media were collected and centrifuged at 500× *g* for 5 min to get rid of cellular debris. The supernatant was then filtrated with a 0.22 µm filter (MilliporeSigma, Burlington, MA, USA). The filtrated conditioned medium was stored in a −80 °C freezer [3].

### 4.2. Differentiation of BMSCs

For osteogenic and adipogenic differentiation, BMSCs expanded in each medium were plated in CellBIND 12-well plates at a density of 3000–4000 cells/cm^2^ and cultured in the respective medium until they reach 90% confluency. Osteogenic and adipogenic differentiation was induced by StemPro Osteogenesis Differentiation Kit (ThermoFisher Scientific, Waltham, MA, USA) and StemPro Adipogenesis Differentiation Kit (ThermoFisher Scientific), respectively. After culturing for 21 days, osteoblastic differentiation was confirmed by calcium deposition visualized by staining with 2% alizarin red S staining (MilliporeSigma). After 14 days induction, adipogenic differentiation was detected by staining lipid droplets with oil red O (MilliporeSigma) [40]. For chondrogenic differentiation, 2 × 10^5^ BMSCs in 20 µL were carefully placed in a well of the 12-well plate. BMSCs were allowed to adhere to the plate at 37 °C for 2 h before chondrogenic induction for 14 days by StemPro Chondrogenesis Differentiation Kit (ThermoFisher Scientific). Chondrogenic differentiation was identified by staining proteoglycan matrix production with Alcian blue at pH 1.0 (MilliporeSigma) [69].

### 4.3. EV Isolation

The filtrated conditioned medium stored at −80 °C was thawed and ultracentrifuged at 110,000× *g*, 4 °C for 2.5 h to obtain EV pellet using Sorvall Discovery 90SE centrifuge (Hitachi, Schaumburg, IL, USA) with AH-629 swinging bucket rotor (ThermoFisher Scientific). The EV pellet was washed with PBS at the same speed for 0.5 h [3].

### 4.4. Flow Cytometry

BMSCs expanded in each medium were harvested and stained with either anti-human PE-CD3 (clone HIT3a), APC-CD11b (M1/70), APC-CD14 (63D3), BV421-CD19 (HIB19), PE-CD29 (MAR4), PE-CD34 (561), BV421-CD45 (HI30), Alexa Fluor 647-CD49e (IIA1), PE-CD73 (AD2), PE-CD90 (5E10), Alexa Fluor 647-CD105 (266), or PE-CD166 (3A6) followed by analysis with a BD LSRII flow cytometer (BD Biosciences, San Jose, CA, USA). The acquired data were processed with FlowJo software (BD, Ashland, OR, USA).

### 4.5. Nanoparticle Tracking Analysis

The size and concentration of EVs in the conditioned medium were measured with a NanoSight NS300 (Malvern Panalytical, Westborough, MA, USA) as previously described [3]. Specifically, the thawed conditioned medium was loaded into the NanoSight NS300 and 30-s video was recorded three times with the setting of screen gain 1 and camera level 14 at 25 °C. The data were analyzed using NTA software 3.3 with the setting of screen gain 13.6 and detection threshold 5.

### 4.6. RNA Isolation

RNA was isolated using mirVana miRNA Isolation Kit (ThermoFisher Scientific) following the manufacturer’s instruction. Specifically, EV pellets isolated from the ultracentrifugation were disrupted with the lysis solution. Following organic extraction using acid-phenol: chloroform, total RNA was isolated and eluted in nuclease-free water.

### 4.7. Small RNA Sequencing

RNA quantity and quality isolated from EVs were evaluated with an Agilent 2100 Bioanalyzer (Agilent Technologies, Santa Clara, CA, USA). Small RNA-sequencing libraries were prepared from 1–250 ng total RNA using the QIAseq miRNA Library Kit (Qiagen, Germantown, MD, USA) as per the manufacturer’s instructions. This system offers a built-in unique molecular identifier (UMI) application, which is used to eliminate possible PCR duplicates in sequencing datasets and, therefore, facilitate unbiased gene expression profiling. The unique barcode sequences were incorporated in the adaptors for multiplexed high-throughput sequencing. The final product was assessed for its size distribution and concentration using BioAnalyzer High Sensitivity DNA Kit (Agilent Technologies). Pooled libraries were diluted to 3 nM in EB buffer (Qiagen) and then denatured using the Illumina protocol. The denatured libraries were loaded onto an S1 flow cell on an Illumina NovaSeq 6000 (Illumina, San Diego, CA, USA) and run for 60 cycles according to the manufacturer’s instructions.

### 4.8. Data Analysis

De-multiplexed sequencing reads were generated using Illumina bcl2fastq (released version 2.20.0.422, Illumina) allowing no mismatches in the index read. Primary read mapping and UMI analysis were conducted via the GeneGlobe Data Analysis Center (Qiagen). DESeq2 R package [70] was used to determine differentially expressed genes by taking into account a paired design where each BMSC sample was compared between DMEM and HPM media. Significance was defined to be those with adjusted *p*-value < 0.1 calculated by the Benjamini–Hochberg method to control the false discovery rate (FDR). The list of differentially expressed genes was analyzed with the miRNA target filter functionality in ingenuity pathway analysis (IPA) to predict the impact of changes in miRNA expression on cellular processes, pathways, diseases, and phenotypes based on its proprietary manually curated database on miRNA-related pathways from published literature.

### 4.9. Statistical Analysis

Statistical analyses were performed with the unpaired two-tailed t-test for comparison of two samples and one-way or two-way analysis of variance for multiple samples followed by Tukey’s multiple comparison test using GraphPad Prism (GraphPad Software, San Diego, CA, USA). Data are shown as mean ± standard error. *p* < 0.05 was determined statistically significant.

## 5. Conclusions

Our study demonstrates that rapidly expanding BMSCs induced by HPM significantly increase EV production; however, the miRNA contents are substantially altered compared to the same BMSCs cultured in the conventional culture medium. These findings suggest that the careful selection of culture medium is critical for scalable and reproducible EV manufacture.

## Data Availability

The sequencing data underlying this article are available in the GEO (GSE185942).
